# Sudden death after valve-in-valve procedure due to delayed coronary obstruction: a case report

**DOI:** 10.1186/s13256-018-1785-x

**Published:** 2018-09-05

**Authors:** Angelo Buscaglia, Giacomo Tini, Gian Paolo Bezante, Claudio Brunelli, Manrico Balbi

**Affiliations:** Clinic of Cardiovascular Disease, San Martino Policlinic Hospital, University of Genova, Largo Rosanna Benzi, 10 1632 Genoa, Italy

**Keywords:** Transcatheter aortic valve implantation, Valve-in-valve, Coronary artery obstruction, Degenerated aortic bioprostheses

## Abstract

**Background:**

Valve-in-valve transcatheter aortic valve implantation for degenerated aortic bioprostheses is an effective option for patients at high risk for redo surgery, even if it may be burdened by complications more common in specific settings, such as, coronary artery obstruction.

**Case presentation:**

We present the case of a Caucasic 84-year-old woman with degeneration of a previously implanted aortic Mitroflow bioprosthesis. She underwent a valve-in-valve transcatheter aortic valve implantation with a CoreValve® bioprosthesis. End-procedure coronary angiography demonstrated maintained perfusion of both coronary arteries. However, few hours later, she experienced sudden cardiac death. An autopsy showed that Mitroflow prosthesis leaflets were higher than the left main coronary ostium, and no other possible cause for the sudden death. Fatality was thus ascribed to left main coronary ostium obstruction due to apposition of the Mitroflow leaflet pushed upward by the late expansion of CoreValve®.

**Conclusions:**

Coronary artery obstruction is a frequently fatal complication which usually presents just after valve implantation, but, as reported in our case, it may also have a delayed presentation. Accurate patient’s selection and intraoperative preventive measures can reduce this eventuality.

**Electronic supplementary material:**

The online version of this article (https://doi.org/10.1186/s13256-018-1785-x) contains supplementary material, which is available to authorized users.

## Background

In recent years, valve-in-valve (VIV) transcatheter aortic valve implantation (TAVI) procedures for correction of degenerated aortic bioprostheses appeared to be a feasible and satisfactory option for patients deemed at high risk for redo surgery [1]. However, the efficacy of this method has been reported to be lower in specific settings. In particular, there are concerns about higher residual transvalvular gradients and rate of coronary artery obstruction [2].

We present a case of sudden death which occurred few hours after a successful VIV intervention. The only possible explanation for the event, as demonstrated by autopsy, appeared to be left main coronary ostium (LMCO) obstruction caused by the previously surgically implanted aortic valve’s leaflets, pushed upward by the expansion of the CoreValve® used for the VIV procedure.

## Case presentation

We report the case of a retired Caucasic 84-year-old woman who required a VIV procedure due to the degeneration of a previously implanted aortic bioprosthesis. Her cardiologic history started in 2006 when she experienced syncope and was then diagnosed as having severe aortic stenosis (mean transvalvular gradient 44 mmHg) and severe mitral regurgitation. She reported no previous clinical events. She underwent aortic valve replacement with a Mitroflow number 21 bioprosthesis and a Carpentier-Edwards Physio mitral annuloplasty ring implantation. After surgery, she suffered from brady-tachy syndrome and needed a pacemaker implantation.

The initial signs of prosthesis degeneration were found at a routine transthoracic echocardiography (TTE) in 2011, with a transvalvular mean gradient of 26 mmHg. However, she was asymptomatic and meanwhile she was diagnosed as having an indolent myeloma, thus a conservative approach was chosen.

In January 2014, she started complaining of epigastric discomfort and dyspnea for minimal exertion. A TTE was repeated and showed a further increase of the mean transvalvular gradient (35 mmHg) and occurrence of moderate paraprosthetic regurgitation due to detachment of the anterior edge of the aortic prosthesis ring.

In February 2014 she was admitted to our department for an episode of pulmonary edema with angina. A physical examination revealed bilateral crackles, 3/6 systolic ejection murmur, and leg swelling. Neurological evaluation was normal. Her electrocardiogram showed transient diffuse ST segment depression and troponin values were slightly elevated (peak, 0.08 ng/ml; reference values, < 0.015 ng/ml). In addition, laboratory tests showed a mild anemia (hemoglobin values, 11 g/dl; reference values, 13–17 g/dl) and a stage 3 chronic kidney disease (serum creatinine, 1.1 mg/dl; reference values, 0.5–1 mg/dl; and glomerular filtration rate, 47 mg/dl). Her hepatic function was normal, as well as white blood cells and platelets count (white blood cells, 6500/ml; reference values, 4500–9800; and platelets count, 300 000/ml; reference values, 150,000–450,000).

A transesophageal echocardiogram (TEE) showed hypomobility of the non-coronary cusp, moderate paraprosthetic regurgitation, and severe intraprosthetic regurgitation. Due to the frailty of our patient, in consideration of age, previous cardiac surgery, and concurrent hematologic disease, after a Heart Team discussion, a VIV TAVI was proposed. Pre-procedural investigations included a coronary angiogram, showing absence of coronary artery disease, and a computed tomography (CT) angiography to calculate the diameter of the Mitroflow prosthesis (17 mm) and the LMCO height from the valvular annulus (11.6 mm).

**Fig. 1:**
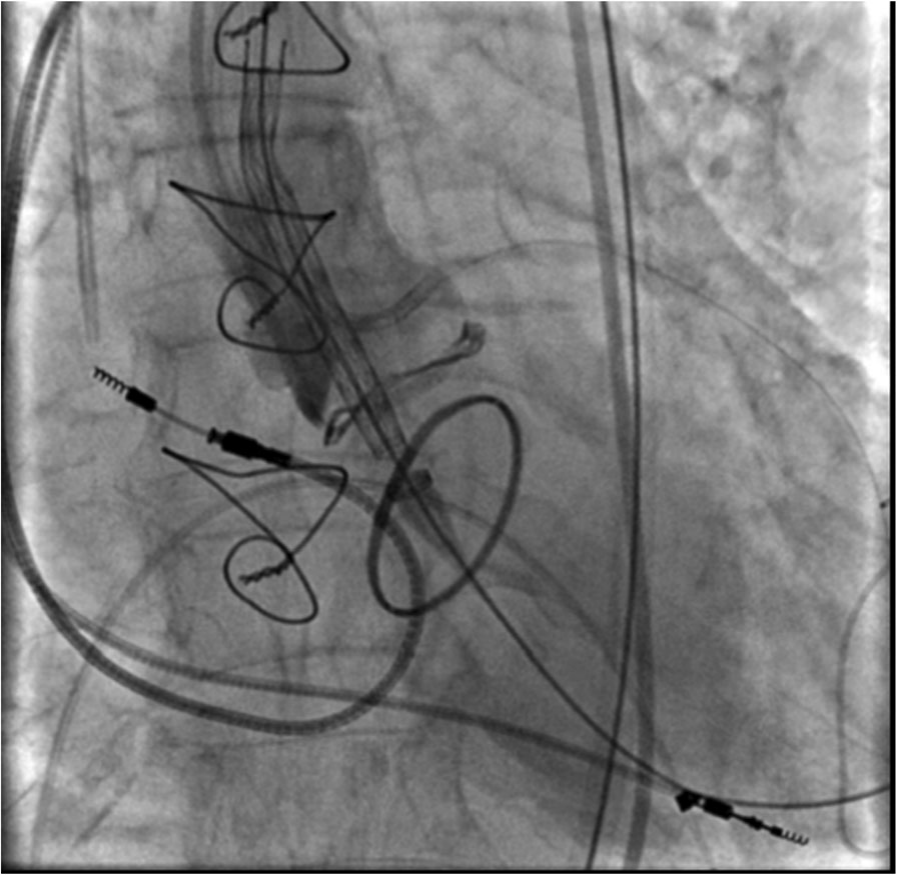
Pre-implantation. Bioprosthesis Mitroflow number 21, Carpentier-Edwards Physio annuloplasty ring, permanent pacemaker, temporary pacemaker, and guide catheter into the left coronary artery

**Fig. 2:**
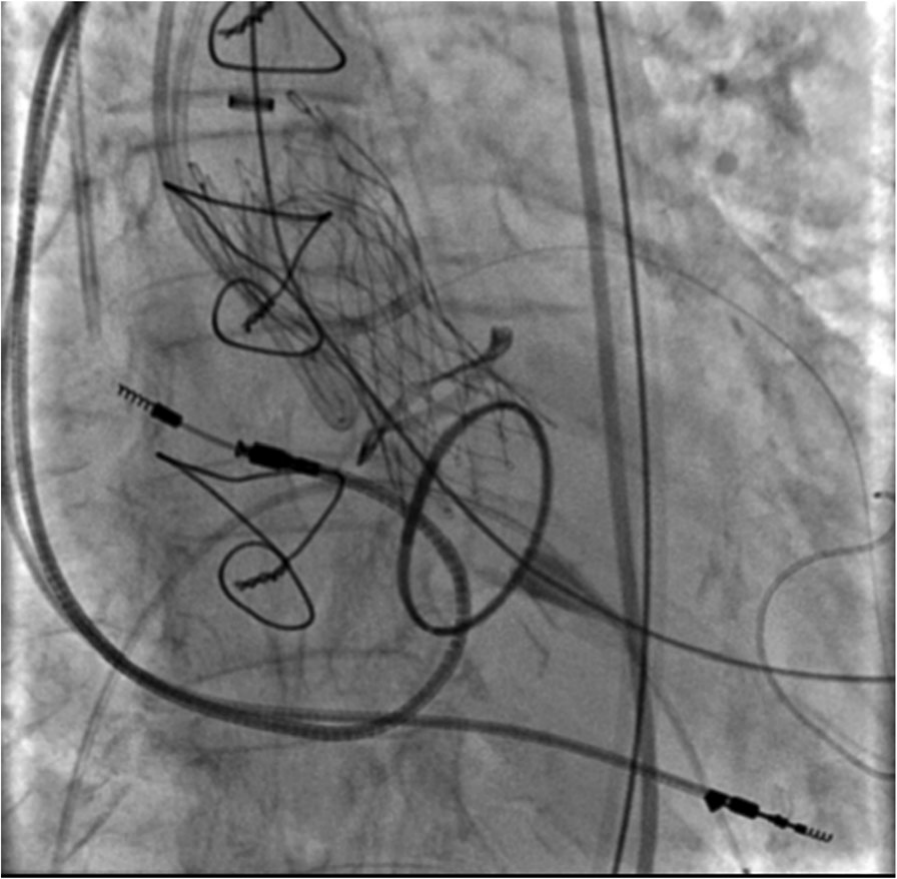
CoreValve® prosthesis number 23 implanted inside the Mitroflow prosthesis number 21

VIV implantation was performed via right femoral artery using a CoreValve® prosthesis number 23 (Additional file 1). A 6 Fr guide catheter, via right omeral artery, was used for the left main coronary artery cannulation as protection (Figs. 1 and 2).

**Fig. 3:**
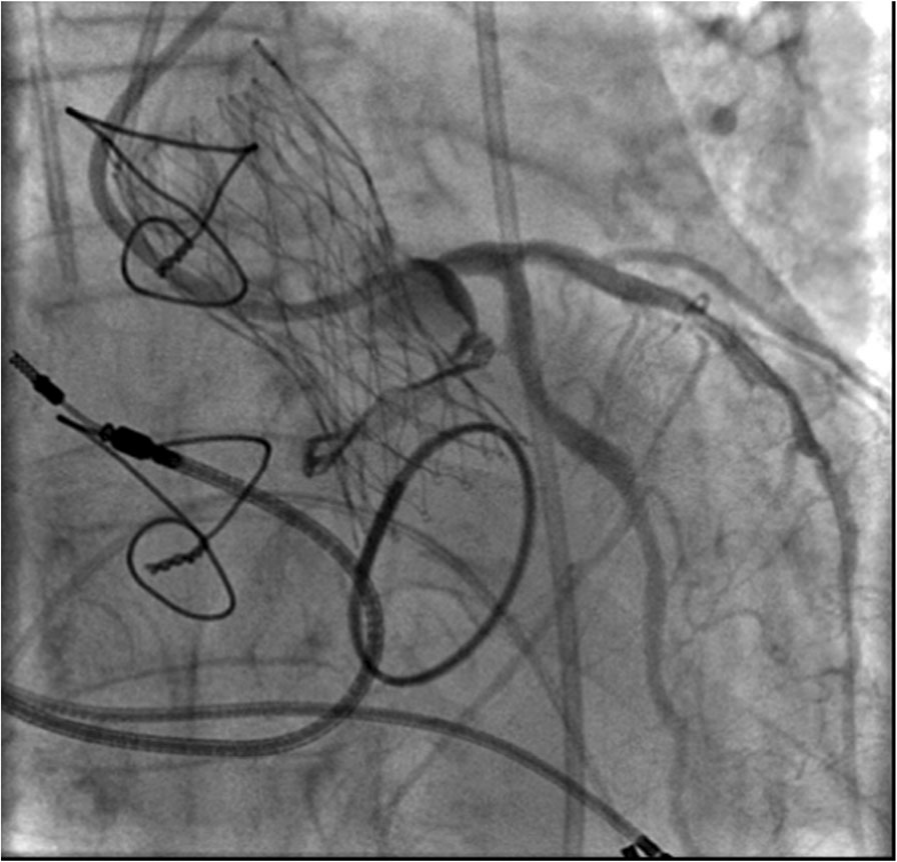
Post-procedural coronary angiography showing normal perfusion of left coronary arteries

**Fig. 4:**
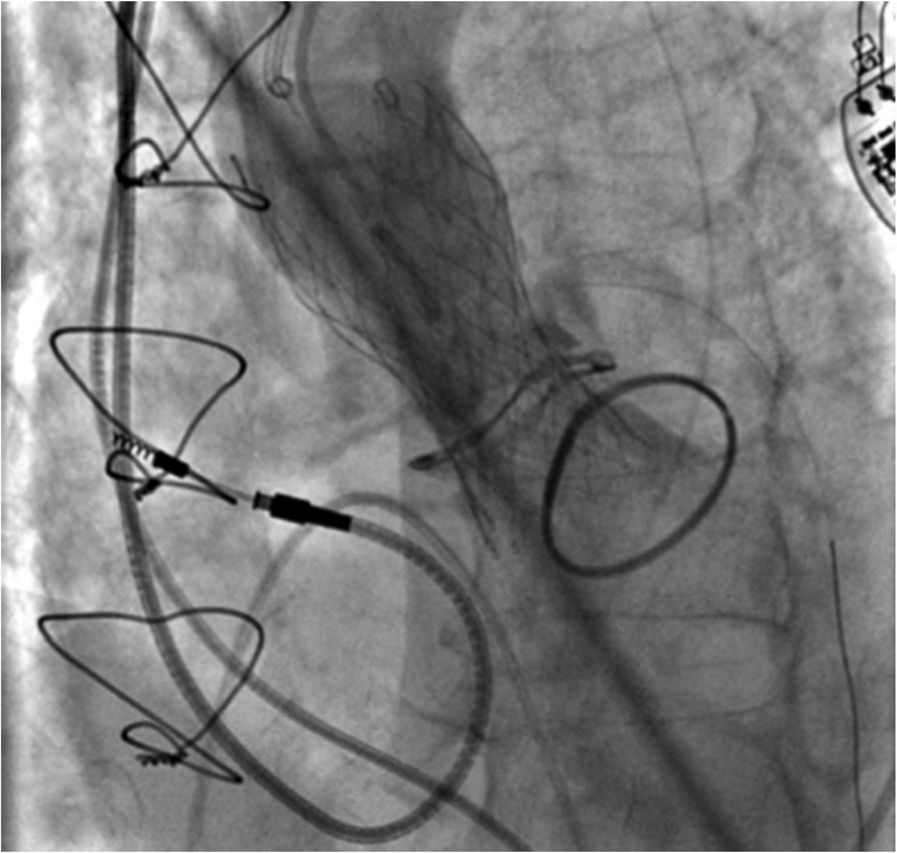
Mild residual aortic regurgitation

Post-procedural angiography confirmed maintained coronary perfusion (Fig. 3), even after the removal of the guide wire. It also showed correct position of the prosthesis and its normal functioning with a transvalvular gradient of 12 mmHg and a mild intraprosthetic regurgitation (Fig. 4).

Our patient was hemodynamically stable and was transferred to the cardiothoracic intensive care unit. Two hours after the end of the procedure, she experienced a sudden cardiac arrest with asystole and electromechanical dissociation. Resuscitation maneuvers were ineffective.

**Fig. 5:**
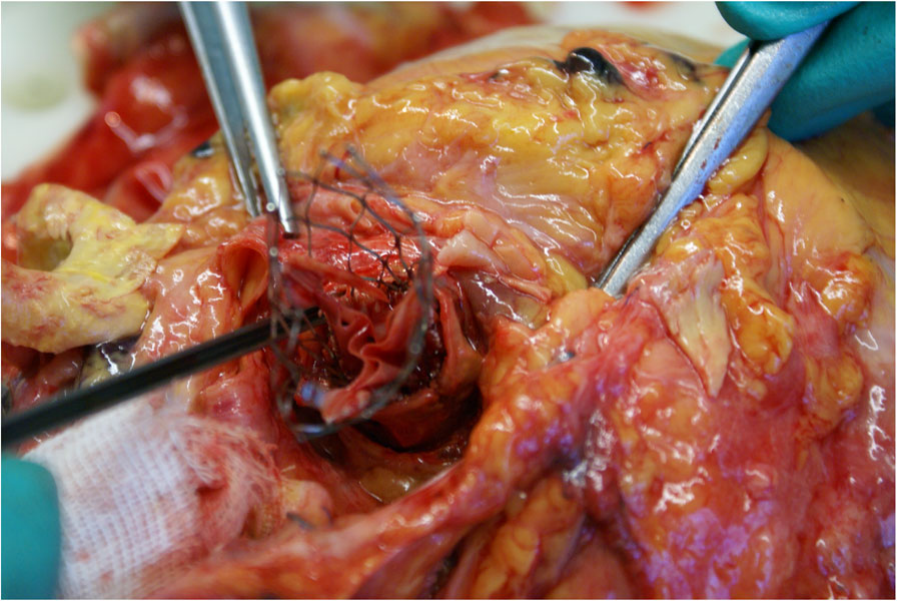
CoreValve® prosthesis removed from the aortic root. Lower left clamp is placed into the left coronary ostium

**Fig. 6:**
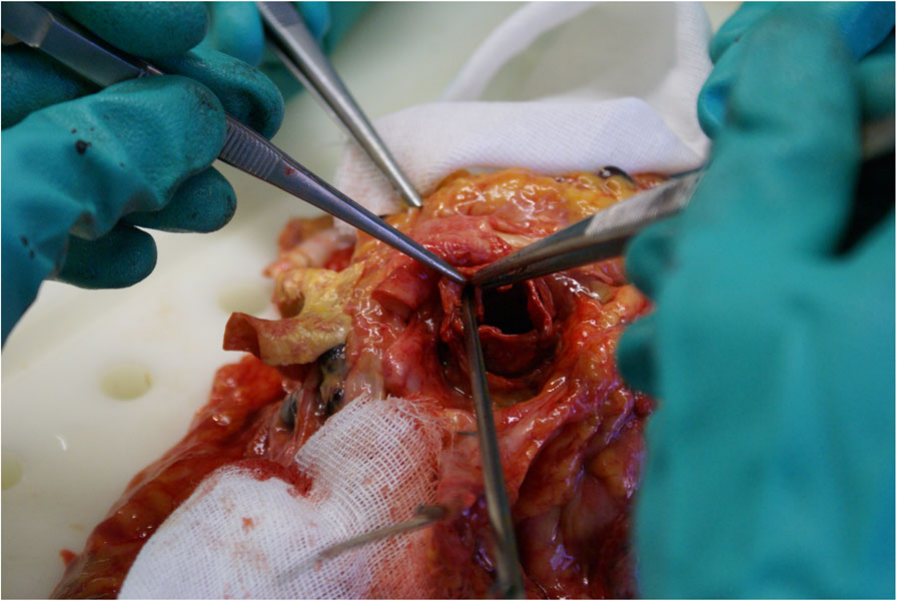
Exposure of the Mitroflow prosthesis. Right clamp holds the bioprosthesis cusp, left clamp holds the aortic root, and lower clamp is placed into the left coronary ostium. Mitroflow leaflets appear higher than the ostium

An autopsy was performed to investigate the cause of death. The CoreValve® prosthesis was removed from the aortic root with no signs of damage or thrombus formation (Fig. 5). The underlying Mitroflow valve appeared free of calcium or thrombi, but its leaflets appeared higher than the LMCO (Fig. 6). No other possible cause for the sudden cardiac death could be found. Our hypothesis was: a delayed occlusion of the LMCO by the Mitroflow leaflets, pushed upward by the late expansion of the CoreValve® prosthesis.

## Discussion

Here we described a case of VIV TAVI complicated by coronary obstruction and sudden cardiac death. Even if the procedure is performed with coronary protection and perfusion is maintained at the end of the intervention, our case shows that this severe adverse event may also occur hours after the end of the TAVI, due to late expansion of the prosthesis.

Use of bioprostheses rather than mechanical valves in surgical aortic valve replacement has increased in recent years [3]. Bioprostheses are characterized by progressive leaflet degeneration, which may ultimately result in valve stenosis, regurgitation, or both, possibly leading to symptomatic heart failure [4, 5]. In the case of bioprosthesis degeneration, even if reoperation is considered the standard treatment, patients are frequently at high risk for a second conventional surgical intervention [6, 7]. VIV TAVI inside the previously surgically implanted failed aortic bioprosthesis has become a reliable alternative to repeat surgery, because it is less invasive and has a high rate of procedural success [1]. Moreover, VIV procedures have lower rates of intervalvular leaks and lower rates of atrioventricular blocks, due to the protection given by the sewing ring of the previously implanted prosthesis that renders a compression of the conduction system less probable [1].

However, the efficacy of VIV procedures has been reported to be lower in specific settings [8, 9]. Worse outcomes are in fact reported for patients with small surgical valves and in those with a stenotic presentation [8]. The poor distensibility of the bioprosthetic ring accounts for elevated residual transaortic mean gradients after VIV TAVI [2]. SAPIEN valves are characterized by annular leaflets and a relation is observed between postoperative gradients after VIV procedure and the size of surgical valve [1]. The same relation is not seen with CoreValve®, due to its supra-annular leaflets that provide a larger orifice [1].

VIV interventions may also be complicated by the life-threatening occurrence of coronary obstruction. The frequency of this severe event is reported to be higher in VIV than in native valve procedures, up to 3.5% versus 0.7% of cases [2]. In a recent series by Jabbour *et al.*, coronary obstructions in TAVI were found to be significantly more common after VIV procedures [9]. Notably, in the study, delayed coronary obstructions were investigated, distinguishing them as early (occurring < 24 hours to 7 days after the procedure) and late delayed (> 7 days). VIV was a risk factor for both.

Coronary obstructions are usually secondary to the displacement of the leaflet of the underlying valve toward a coronary ostium, most commonly the left one [9–12], as in the case presented.

In addition to the anatomic features known to possibly predispose to coronary obstruction in the case of native valve procedures, namely shallow sinus of Valsalva or low-lying coronary ostium [9, 10], in VIV the risk is also dependent on the characteristics of the previously implanted surgical bioprosthesis and on the relationship of its leaflets and posts with the coronary ostia [2].

Coronary obstructions are reported to be more common with stenotic than with regurgitant bioprostheses, supra-annular or internally stented bioprostheses, bulky bioprosthetic leaflets and reimplanted coronary arteries [12]. Internally stented bioprostheses, such as Mitroflow, have relatively long leaflets that may extend outward in a tubular fashion after a VIV implantation [12]. Stentless bioprostheses are usually implanted in a supra-annular position, thus the valve leaflets are closer to the coronary ostia [13].

If there is suspicion of coronary obstruction an immediate angiography must be carried out, and if diagnosis is confirmed, percutaneous coronary intervention (PCI) should be considered or in the case of PCI failure, emergent coronary artery bypass should be considered [12]. In a patient with high risk for coronary obstruction additional preventive measures are advised, such as coronary protection with a guidewire, leaving an undeployed stent in the distal portion of the coronary ready to be pulled back and implanted in the ostium if needed [10, 11]. In selected cases, a preventive stent implantation with chimney technique, even in a case of maintained perfusion of the coronary, may be reasonable. In our center, after the case described here, this technique was successfully performed in a subsequent case of a VIV TAVI on a previous Mitroflow number 21 prosthesis dysfunction. In addition, the use of a retrievable valve may permit rapid prosthesis removal in the case of coronary obstruction evidence [12]. The selection of a smaller sized valve provides less displacement of bioprosthesis leaflets than the low implantation does [1].

Coronary obstructions after TAVI may be acute or delayed, as in the case presented (early delayed, according to Jabbour *et al*. [9]). No other cause for the sudden death experienced by our patient except for LMCO obstruction was demonstrated by an autoptic examination. It must, in fact, be considered that an implanted bioprosthesis may reach full expansion only in the first hours after the completion of the VIV procedure; thus, displacement of the underlying valve’s leaflets may not be immediate [9, 13]. This mechanism is particularly true for CoreValve® prostheses [13]. Notably, the bioprosthesis our patient received in 2006 was a Mitroflow number 21. Other authors have previously underlined the need to cautiously execute or even to avoid VIV procedures on Mitroflow bioprostheses [14].

A delayed occurrence of coronary obstruction renders this complication, already burdened by an extremely high mortality, even more difficult to manage. Therefore, an adequate and careful assessment of patients who receive indication for a VIV procedure is of paramount importance. Even though rare, in fact, some complications are sudden and fatal. Since VIV procedures have shown satisfactory results, the best way to limit unsuccessfulness is prevention of complications by accurate pre-selection of patients.

## Conclusions

VIV TAVI for previously implanted degenerated aortic bioprostheses is a satisfactory alternative method to the repeating of surgery, it is less invasive and has a high rate of procedural success. However, coronary obstruction, a life-threatening complication, seems to occur more frequently in VIV than in native valve TAVI.

This complication usually occurs at the time of implantation, but, as reported in our case, it may also have a delayed presentation. This highlights the importance of adequate preoperative selection of patients as a means of prevention.

## Additional file

Additional file 1: Developing CoreValve®. (MP4 3635 kb)

## Abbreviations

CT: Computed tomography
LMCO: Left main coronary ostium
PCI: Percutaneous coronary intervention
TAVI: Transcatheter aortic valve implantation
TEE: Transesophageal echocardiogram
TTE: Transthoracic echocardiography
VIV: Valve-in-valve
